# Association of Common Variants in HNF1A Gene with Serum AFP Level in Healthy Chinese Individuals and HCC Patients

**DOI:** 10.1155/2019/6273497

**Published:** 2019-11-07

**Authors:** Xue-jun Li, Dong-hua Shao, Mei-lin He, Guo-wei Liang

**Affiliations:** Department of Clinical Laboratory, Aerospace Center Hospital, No. 15 Yuquan Road, Haidian District, Beijing 100049, China

## Abstract

Although alpha-fetoprotein (AFP) is a widely used tumor marker in hepatocellular carcinoma (HCC), 40% of newly diagnosed patients do not have an elevated AFP level. Research has revealed that mutations in the HNF1A binding site of the AFP gene promoter cause significantly elevated serum AFP levels in patients with hereditary persistence of AFP. This study investigated the relationship between HNF1A genetic variants and serum AFP levels. We examined the association between the HNF1A-rs1169288 (A/C), rs2464196 (G/A), and rs1169310 (C/T) polymorphisms and AFP levels in a healthy Chinese population (*n* = 1010) and HCC patients (*n* = 185). Single nucleotide polymorphisms were genotyped by the amplification refractory mutation system combined with TaqMan probe in real-time PCR. The serum AFP concentrations were measured using the Architect i2000 immunochemistry analyzer. In healthy individuals, serum AFP levels were significantly lower with the rs2464196-AA and rs1169310-TT genotypes. Similar significant differences were observed in HCC patients. Moreover, in HCC patients, the distribution frequencies of rs2464196-AA+AG and rs1169310-TT+TC among those with AFP ≤ 20 ng/ml or ≤400 ng/ml were significantly lower than those in patients with AFP > 20 ng/ml or >400 ng/ml. Among all subjects, those carrying the HNF1A-rs2464196-A or rs1169310-T allele tended to have low levels of AFP. However, the HNF1A-rs1169288 polymorphism showed no significant association with the serum AFP level. These findings provide new insight into the genetic determinants of serum AFP level and can aid the differential diagnosis of HCC patients with low serum AFP.

## 1. Introduction

Hepatocellular carcinoma (HCC), one of the most frequently occurring cancers worldwide, typically develops on a basis of chronic liver disease and is associated with a short survival time largely due to the limited treatment options [1]. Alpha-fetoprotein (AFP) is a well-recognized tumor marker of HCC; however, an elevated serum AFP level is only found in approximately 60% of all newly diagnosed HCC patients [2]. This phenomenon creates confusion for the diagnosis and treatment monitoring of HCC patients. Currently, the exact mechanism through which AFP levels are not significantly elevated in about 40% of HCC patients remains unclear. In addition, the serum AFP concentration is also elevated in pregnancy, acute or chronic hepatitis, and liver cirrhosis as well as in cases of alcohol- or drug-induced liver damage [3, 4].

Previous studies have demonstrated that point mutations in the hepatocyte nuclear factor 1 homeobox A (HNF1A) binding sites of the AFP gene promoter can result in elevated serum AFP levels in related patients with hereditary persistence of AFP (HPAFP) without pathological conditions [5–7]. These mutations could increase the ability of HNF1A to bind to the AFP promoter and enhance AFP transcriptional activity, thus leading to the increased level of AFP in serum [5, 6]. These findings highlight the importance of the HNF1A binding site in the regulation of the AFP gene and also suggest that the serum AFP concentration may be influenced by mutations of the HNF1A gene. To date, it has not been reported whether HNF1A gene variants are associated with the serum AFP level in a general population.

Although the regulatory mechanism of AFP expression is complicated, prior studies have suggested that the transcription process is the determining step for AFP gene regulation [8–10]. The AFP gene has a 7 kb upstream regulatory region that includes a tissue-specific promoter, three independent enhancers, and a silencer [11], and HNF1A is the key regulator of AFP gene promoter expression [5–7, 11]. Previous studies showed that AFP promoter mutations in the distal HNF1-binding region and the proximal HNF1-binding region play important roles in regulating AFP expression [12, 13]. Currently, HNF1A gene variants are associated with maturity onset diabetes of the young (MODY) [14, 15], C-reactive protein (CRP) levels [16–18], gamma-glutamyl transferase (GGT) levels [19, 20], total cholesterol (TC) levels [21], pancreatic cancer [22], coronary artery disease [21, 23], and metabolic syndrome (MS) [24]. The most common variants in HNF1A are rs1169288 (A/C, Ile27Leu), rs2464196 (G/A, Ser487Asn), and rs1169310 (C/T), which have been reported to be associated with the CRP level, coronary artery disease, and diabetic retinopathy [17, 18, 25, 26].

Considering these previous findings, we hypothesized that genetic variants of the HNF1A gene are associated with serum AFP levels. To test this hypothesis, we selected common polymorphisms of HNF1A (rs1169288, rs2464196, and rs1169310) as candidate single nucleotide polymorphisms (SNPs) and examined their association with the serum AFP levels in a Chinese healthy population and in newly diagnosed HCC patients.

## 2. Materials and Methods

### 2.1. Study Subjects

From January 2018 to November 2018, 1010 healthy individuals (615 males and 395 females) aged 25–65 years were enrolled from Aerospace Central Hospital (Beijing, China), and 185 newly diagnosed and pathologically confirmed HCC cases aged 32–84 years were recruited from Aerospace Central Hospital, Peking University Cancer Hospital, and Cancer Hospital Chinese Academy of Medical Sciences. All participants were unrelated and ethnically Han Chinese. Written informed consent was obtained from all participants. This study was approved by the ethics committee of the Aerospace Central Hospital and conducted following the principles of the Declaration of Helsinki.

Healthy individuals were excluded if they met any of the following criteria: (1) acute or chronic viral hepatitis infection (a positive test for hepatitis B virus surface antigen and/or hepatitis C virus antibody), (2) alcohol consumption ≥ 140 g/week for men or ≥70 g/week for women, (3) pregnancy, (4) liver cancer, or (5) other malignancies. The maximum AFP in our 1010 apparently healthy individuals was 18.43 ng/ml. Therefore, we assumed that individuals with HPAFP were excluded as HPAFP is a rare hereditary disease and HPAFP patients carrying AFP promoter mutations (-119G>A and -55C>A) have serum AFP levels significantly greater than 20 ng/ml [5–7].

The overall exclusion criteria for HCC cases were major cardiac rhythm disturbances, major psychiatric illness or substance abuse, history of cerebral vascular disease, incapacitating or life-threatening illness, neurological disorder, and administration of psychotropic medication.

### 2.2. Anthropometric and Biomarker Measurements

For healthy individuals, a questionnaire was administered and anthropometric measurements were taken during their regular healthy examination. The following data were obtained for each person: age, sex, body mass index (BMI), and blood pressure. BMI was calculated as body weight (kg)/height^2^ (m^2^). Systolic and diastolic blood pressure was measured three times after a 10 min rest, and the mean value was used in subsequent analyses. Fatty liver disease was diagnosed based on abdominal ultrasonography (IU-22, Philips, Bothell, USA) performed by a skilled sonographer [27].

For HCC patients, tumor grading and staging were determined after surgery according to the criteria of the World Health Organization and the Tumor Lymph Node Metastasis (TNM) classification of the International Union Against Cancer based on the tumor number, size, vascular invasion, lymphatic involvement, and metastasis [28, 29].

A 3 ml sample of fasting venous blood was collected from each healthy individual and from each newly diagnosed HCC patient before surgery. The serum was separated within 2 hours, stored at -80°C, and used for analysis within 3 months. In fasting venous blood samples, the alanine aminotransferase (ALT), aspartate aminotransferase (AST), GGT, fasting plasma glucose (FPG), triglycerides (TG), TC, low-density lipoprotein cholesterol (LDL-C), and high-density lipoprotein cholesterol (HDL-C) concentrations were measured using an automated chemistry Analyzer (AU 2700, Olympus Co., Tokyo, Japan). CRP levels were determined using an immunonephelometry assay (Dade Behring, Newark, NJ, USA). Serum AFP, hepatitis B virus surface antigen, and hepatitis C virus antibody levels were measured using the American Abbott i2000 Immunoassay Analyzer (Abbott Co., Santa Clara, CA, USA). The supporting reagents as well as calibrator and quality control materials were provided by Abbott Co. The intra- and interassay coefficients of variation (CVs) for AFP measurement were 3.5%–4.7% and 4.1%–8.1%, respectively.

### 2.3. Genomic DNA Extraction

For each participant, genomic DNA was extracted from 200 *μ*l EDTA-preserved whole blood using a Tiangen DNA isolation Kit (Tiangen Biotech Co., Ltd., Beijing, China) according to the manufacturer's protocol. After extraction, the genomic DNA samples were immediately stored at −80°C.

### 2.4. SNP Selection and Genotyping

SNPs were selected based on the following principles. SNPs are preferred if they are exon missense mutations with a minor allele frequency (MAF) > 5% (1000 Genomes database) or they have been reported in studies related to certain traits or diseases [17, 18, 25, 26]. The following three SNPs were selected: rs1169288, rs2464196, and rs1169310.

Genotyping for all three HNF1A SNPs was performed using the amplification refractory mutation system (ARMS) combined with TaqMan probe in a real-time polymerase chain reaction (PCR) [30, 31]. The assay mixture included the allele-specific primer, a common primer, and TaqMan probe. The 3′ ends of the two allele-specific primers were wild bases and mutant bases. They were used to form two fluorescent quantitative PCR reaction systems with common primers and TaqMan probe. An additional mismatch was designed at the 3′ end of all allele-specific primers in the 2 positions in order to strengthen the allelic-specific discrimination (Supplementary [Supplementary-material supplementary-material-1]). Genotyping results were analyzed based on the *Δ* cycle threshold (*Δ*Ct, ΔCt = Ct value of wild allele reaction–Ct value of mutation allele reaction).

All PCR reactions were performed with a Tiangen Real Master Mix (Probe) kit (Tiangen Biotech Co., Ltd., Beijing, China) on an ABI Prism sequence detection system 7500 (Applied Biosystems, Foster City, CA, USA). The primers and probes were designed using primer premier 5.0 software (Premier Biosoft International, Palo Alto, CA, USA) and synthesized by Beijing Semiconductor Technology Corp. (Beijing, China). The final volume for each PCR was 20 *μ*l and included 1 *μ*l forward primer (0.25 *μ*M), 1 *μ*l reverse primer (0.25 *μ*M), 1 *μ*l TaqMan probe (0.25 *μ*M), 8 *μ*l 2x real master mix, 1 *μ*l enhance solution, 6 *μ*l ddH_2_O, and 2 *μ*l DNA template. All reactions were run at 95°C for 2 min, 95°C for 15 s, and 60°C for 1 min for 40 cycles. The genotyping success rate was 100% for all SNPs. Additionally, 30 samples were randomly selected from our study subjects and regenotyped by direct sequence analysis using a commercial sequencing service (SinoGenoMax, Beijing, China) for the purpose of validating the accuracy of our PCR genotyping method. The comparison results showed a 100% match between the two assays (Supplementary [Supplementary-material supplementary-material-1]).

### 2.5. Statistical Analysis

All statistical analyses were performed using the SPSS 20.0 statistical package (SPSS Inc., Chicago, IL, USA). Hardy-Weinberg equilibrium was assessed using the Chi-squared test. Linkage disequilibrium (LD) between polymorphisms was estimated using Haploview. Among each SNP genotype, differences were evaluated for categorical variables using a Chi-squared test and for quantitative variables using one-way analysis of variance (ANOVA). The TG, CRP, GGT, ALT, AST, and AFP data were natural logarithm-transformed before statistical analysis to adhere to a normality assumption.

The associations of rs2464196 and rs1169310 genotypes with serum AFP levels were analyzed by a linear regression model, and all variables that might affect serum AFP levels were used as covariates in the healthy group or HCC group, respectively. Correction for multiple testing was performed using Bonferroni's method. SNPs with a nominal *P* value < 0.0167 were considered significant.

## 3. Results

### 3.1. Part 1: Association of HNF1A SNPs with Serum AFP Level in Healthy Subjects

#### 3.1.1. Basic Characteristics and NHF1A SNP Distribution Frequencies among Healthy Chinese Individuals

The basic characteristic and HNF1A SNP distribution frequencies among 1010 healthy Chinese Han subjects, including 615 males and 395 females, are presented in Table 1. No significant differences were observed in age, LDL-C level, and AFP concentration between males and females (*P* > 0.05). Compared to the female subjects, the male subjects had a significantly higher BMI, systolic pressure, diastolic pressure, and AST, ALT, GGT, TG, CRP, and FPG levels as well as a higher incidence of fatty liver disease (*P* < 0.05), but the male subjects also had significantly lower HDL-C and TC levels (*P* < 0.05).

The 1010 healthy individuals were divided into three groups based on the genotyping results. Only the CRP level (*P* = 0.023) differed significantly among the genotypes of rs1169288, as there were no significant differences in gender, age, BMI, blood pressure, LDL, HDL, TC, AST, FPG, ALT, TG, GGT, or fatty liver disease among the genotypes of each SNP locus of HNF1A (*P* > 0.05; Supplementary [Supplementary-material supplementary-material-1]). The MAFs for rs1169288-C, rs2464196-A, and rs1169310-T in this healthy population were 0.453, 0.479, and 0.484, respectively. No significant gender differences were observed in the allele frequencies of all SNPs (*P* > 0.05). The allele frequencies of all three SNPs were in Hardy-Weinberg equilibrium in each group (*P* > 0.05), and all three SNPs were in significant LD (*R*^2^ range, 0.385–0.937; *P* < 0.001). Moreover, rs1169310 and rs2464196 were almost in complete LD (*R*^2^ = 0.937, *P* < 0.001).

#### 3.1.2. Differences in Serum AFP Level with Different Genotypes according to the Studied HNF1A SNPs in the Healthy Population

**Figure 1:**
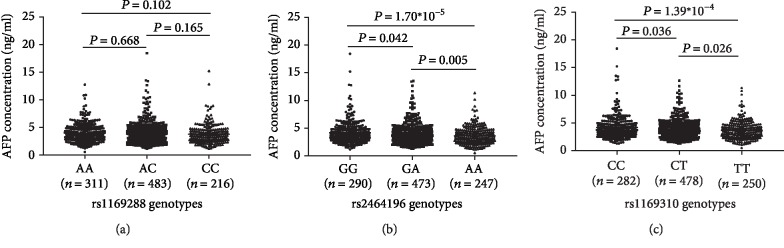
Serum AFP levels in healthy Chinese subjects (*n* = 1010) with different HNF1A SNP genotypes. The scatter plot shows the mean AFP level (±standard deviation (SD)) stratified by SNP genotype. The AFP data were ln-transformed before analysis. *P* values were calculated by ANOVA. (a) AFP levels did not differ significantly among the rs1169288 genotypes (*P* = 0.238); (b) significant differences of AFP levels among the rs2464196 genotypes (*P* < 0.001); (c) significant differences of AFP levels among the rs1169310 genotypes (*P* = 0.001).

As shown in Figure 1, significant differences in serum AFP levels were observed among the genotypes of rs2464196 (*P* < 0.001) and rs1169310 (*P* = 0.001). Analysis of the basal AFP level in individuals with the different rs2464196 and rs1169310 genotypes both demonstrated gene dose-specific effects on the AFP level. The mean serum AFP level was 3.60 ± 1.59 ng/ml in the rs2464196-AA group (lowest), 3.92 ± 1.78 ng/ml in the AG group (middle), and 4.21 ± 2.10 ng/ml in the GG group (highest). Compared with those in individuals carrying the rs2464196 GG allele, the average serum AFP levels in individuals carrying the AA allele and GA+AA allele were lower by 14.5% (*P* < 0.001) and 9.5% (*P* = 0.001), respectively. The mean serum AFP levels for healthy individuals carrying rs1169310-TT, CT, and CC were 3.67 ± 1.67 ng/ml (lowest), 3.89 ± 1.69 ng/ml (middle), and 4.23 ± 2.19 ng/ml (highest), respectively. Compared with those in individuals carrying the rs1169310 CC allele, the average serum AFP levels in healthy subjects carrying the TT allele and CT+TT allele were lower by 13.2% (*P* < 0.001) and 9.8% (*P* = 0.002), respectively. No significant differences in serum AFP levels were found among individuals carrying the different rs1169288 alleles in the healthy population (3.98 ± 1.70 ng/ml, 3.96 ± 1.94 ng/ml, and 3.78 ± 1.82 ng/ml with rs1169288-AA, AC, and CC, respectively, *P* = 0.238).

#### 3.1.3. Association of rs2464196 and rs1169310 Genotypes with AFP Levels in Healthy Subjects

As shown in Table 2, using AFP as a dependent variable, linear regression analyses of different models showed that the rs2464196 and rs1169310 genotypes were independently associated with an altered serum AFP level in all models (all *P* < 0.0167).

### 3.2. Part 2: Association of HNF1A SNPs with Serum AFP Level in HCC Patients

#### 3.2.1. Basic Characteristics and HNF1A SNP Distribution Frequencies among HCC Patients

Of the 185 HCC patients included in this study, 159 were male and 26 were female. This group included 79 patients (42.7%) older than 60 years, 153 (82.7%) patients with HBV infection, 24 patients (13.0%) with HCV infection, 68 patients (36.8%) with an AFP level < 20 ng/ml, and 118 patients (63.8%) with an AFP < 400 ng/ml. The results of pathological analysis showed that 64 patients (34.6%) had a tumor > 5 cm in diameter. The clinical stage of HCC was classified as I, II, III, and IV in 32, 91, 29, and 33 cases, respectively. Additionally, 35, 107, and 43 cases were categorized as high, middle, and low differentiations, respectively (Table 3).

The numbers of HCC patients carrying the AA, AC, and CC alleles of rs1169288 were 58 (31.4%), 95 (51.3%), and 32 (17.3%), respectively, and the MAF of rs1169288-C was 0.430. The numbers of HCC patients carrying the GG, GA, and AA alleles of rs2464196 were 42 (22.7%), 98 (53.0%), and 45 (24.3%), respectively, and the MAF of rs2464196-A was 0.508. The numbers of patients carrying the CC, CT, and TT alleles of rs1169310 were 40 (21.6%), 101 (54.6%), and 44 (23.8%), respectively, and the MAF of rs1169310-T was 0.511. Of the 185 HCC patients, the allele distribution frequencies of all three SNPs were consistent with the Hardy-Weinberg equilibrium test (rs1169288: *χ*^2^ = 0.42, *P* = 0.81; rs2464196: *χ*^2^ = 0.66, *P* = 0.72; rs1169310: *χ*^2^ = 1.58, *P* = 0.45).

#### 3.2.2. Differences in the Serum AFP Level with Different Genotypes according to the Studied HNF1A SNPs

**Figure 2:**
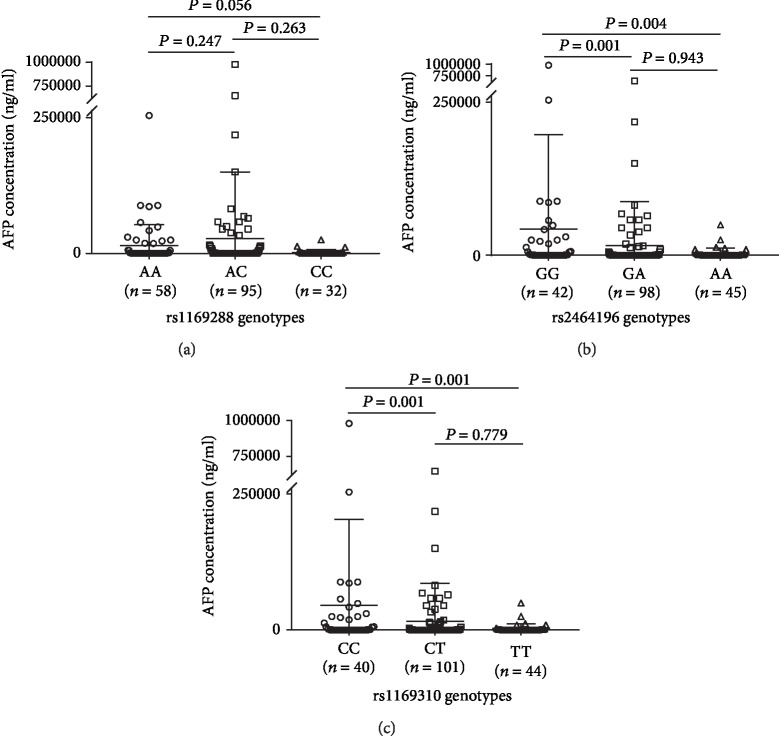
Serum AFP levels in Chinese HCC patients (*n* = 185) with different HNF1A SNP genotypes. The scatter plot shows the mean AFP level (±standard deviation (SD)) stratified by SNP genotype. The AFP data were ln-transformed before analysis. *P* values were calculated by ANOVA. (a) AFP levels differed significantly among the rs1169288 genotypes (*P* = 0.154); (b) AFP levels differed significantly among all of the rs2464196 genotypes (*P* = 0.003), but not between rs2464196-GA and rs2464196-AA (*P* = 0.943); (c) AFP levels differed significantly among all of the rs1169310 genotypes (*P* = 0.001), but not between rs1169310-CT and rs1169310-TT (*P* = 0.779).

As shown in Figure 2, significant differences in serum AFP levels were observed among the genotypes of rs2464196 (*P* = 0.003) and rs1169310 (*P* = 0.001), and these differences indicated gene dose-specific effects. The mean serum AFP levels of HCC patients carrying the rs2464196-AA, rs2464196-AG, and rs2464196-GG alleles were 2935.81 ± 8525.96 ng/ml (lowest), 16340.80 ± 71302.72 ng/ml (middle), and 42854.32 ± 154494.82 ng/ml (highest), respectively. Compared with that among patients carrying the rs2464196-GG allele, the average serum AFP levels of patients carrying the AA allele and GA+AA allele were lower by 93.1% (*P* = 0.004) and 71.7% (*P* = 0.001), respectively. The mean serum AFP levels of HCC patients carrying the rs1169310-TT, rs1169310-CT, and rs1169310-CC alleles were 2708.15 ± 8485.04 ng/ml (lowest), 15985.87 ± 70262.45 ng/ml (middle), and 44991.50 ± 158095.87 ng/ml (highest), respectively. Compared with those of patients carrying the rs1169310-CC allele, the average serum AFP levels of HCC patients carrying the TT allele and CT+TT allele were lower by 94.0% (*P* = 0.001) and 73.4% (*P* < 0.001), respectively. Together, these results suggest that HCC patients carrying the rs2464196-A or rs1169310-T mutant alleles were less likely to have an elevated AFP level. As in the healthy subject group, no significant differences in serum AFP levels were found among HCC patients carrying the different rs1169288 alleles (*P* = 0.154).

#### 3.2.3. Association of rs2464196 and rs1169310 Genotypes with AFP Level

As shown in Table 4, using AFP as a dependent variable, linear regression analyses of different models showed that the rs2464196 and rs1169310 genotypes were independently associated with an altered serum AFP level in all models (all *P* < 0.05), although only the *P* values for model 1 and model 2 could pass Bonferroni correction (*P* < 0.0167).

#### 3.2.4. Comparison of rs2464196 and rs1169310 Genotype Frequencies according to Different AFP Concentration Cut-Off Values

The normal range of AFP is ≤20 ng/ml, and the cut-off of AFP > 400 ng/ml is typically regarded as the diagnostic threshold for HCC [32].  Table 5 presents the rs2464196 and rs1169310 genotype frequencies among HCC patients with a serum concentration of AFP ≤ 20 ng/ml or >20 ng/ml. The results indicated that significant differences were observed among both the rs2464196 and rs1169310 genotype groups (*P* = 0.024 and *P* = 0.017, respectively). The frequency of the rs2464196-AA+AG genotypes was significantly greater in the AFP ≤ 20 ng/ml group than in the AFP > 20 ng/ml group (88.2% vs. 70.9%, *P* = 0.007), and the frequency of rs2464196-GG was significantly lower (11.8% vs. 29.1%, *P* = 0.007), suggesting that HCC patients carrying the rs2464196-A mutant allele were more likely to have an AFP concentration ≤ 20 ng/ml. Similarly, the frequency of the rs1169310-TT+CT genotypes was significantly greater in the AFP ≤ 20 ng/ml group than in the AFP > 20 ng/ml group (89.7% vs. 71.8%, *P* = 0.004), and the rs1169310-CC frequency was significantly lower (10.3% vs. 28.2%, *P* = 0.004), suggesting that HCC patients carrying the rs1169310-T mutation allele also were more likely to have an AFP concentration ≤ 20 ng/ml.

Table 6 presents the rs2464196 and rs1169310 genotype frequencies among HCC patients with a serum AFP concentration > 400 ng/ml or ≤400 ng/ml. Significant differences were observed among both the rs2464196 and rs1169310 genotype groups (*P* = 0.036 and *P* = 0.018, respectively). The frequency of the rs2464196-AA+AG genotypes was significantly greater in the AFP ≤ 400 ng/ml group than in the AFP > 400 group (83.1% vs. 67.2%, *P* = 0.013), and the frequency of rs2464196-GG was significantly lower (16.9% vs. 32.8%, *P* = 0.013), suggesting that HCC patients carrying the rs2464196-A mutant allele were more likely to have a serum AFP concentration ≤ 400 ng/ml. Similarly, the frequency of the rs1169310-TT+CT genotypes was significantly greater in the AFP ≤ 400 ng/ml group than in the AFP > 400 ng/ml group (84.7% vs. 67.2%, *P* = 0.005), and the rs1169310-CC frequency was significantly lower (15.3% vs. 32.8%, *P* = 0.005), suggesting that HCC patients carrying the rs1169310-T mutation allele also were more likely to have a serum AFP concentration ≤ 400 ng/ml.

With AFP ≤ 20 ng/ml as the cut-off value, the number of people carrying the rs1169310-T or rs2464196-A alleles among the 68 HCC patients who did not have an elevated AFP level was 61, accounting for 89.71% of HCC patients without elevated AFP levels. With AFP ≤ 400 ng/ml as the cut-off value, in 118 HCC patients with nonelevated AFP levels, the number of people carrying the rs1169310-T or rs2464196-A allele was 100, accounting for 84.75%. In other words, approximately 89.71% (with AFP ≤ 20 ng/ml as the cut-off value) or 84.75% (with AFP ≤ 400 ng/ml as the cut-off value) of HCC cases without elevated AFP levels could be explained by the HNF1A genotypes.

### 3.3. Part 3: Frequency of HNF1A Haplotypes and Association with AFP Level

SNP regression analysis demonstrated that two sites within the HNF1A gene were significantly associated with the AFP level. Further haplotypes were inferred to capture possible allelic associations. Four major haplotypes were observed (Supplementary [Supplementary-material supplementary-material-1]). In healthy individuals, the haplotype frequencies of GC, GT, AC, and AT of rs2464196 and rs1169310 were 50.98%, 1.15%, 0.60%, and 47.27%, respectively. Because the low haplotype frequencies of GT and AC may affect the parameter estimation in the multiple linear regression model, the GT and AC haplotypes were combined into one category labeled as “other.” As shown in Supplementary [Supplementary-material supplementary-material-1], only the A-T haplotype was significantly associated with a low serum AFP level in all three models (all *P* < 0.001).

In HCC patients, the haplotype frequencies of GC, GT, AC, and AT of rs2464196 and rs1169310 were 48.65%, 0.54%, 0.27%, and 50.54%, respectively. Also, due to their low frequencies, the GT and AC haplotypes were combined into one category (“other”). As presented in Supplementary [Supplementary-material supplementary-material-1], only the A-T haplotype was significantly associated with a low serum AFP level in all the three models (*P* = 0.005, *P* = 0.012, and *P* = 0.049, respectively).

## 4. Discussion

In this study, by examining the associations between genetic variants in the HNF1A gene and serum AFP levels, we found that the HNF1A rs2464196 and rs1169310 polymorphisms were strongly associated with the serum AFP concentration in both the healthy Chinese Han population and Chinese Han HCC patients. Our results indicated that individuals carrying the rs2464196-A allele or rs1169310-T allele were less likely to have an elevated serum AFP level, and the minor alleles of the HNF1A rs2464196 and rs1169310 genotypes as well as the haplotypes A-T were associated with significantly lower AFP levels compared with the other alleles and haplotypes. However, the HNF1A rs1169288 polymorphism showed no significant association with the serum AFP level in either healthy subjects or HCC patients.

The HNF1A gene is located on chromosome 12q24.31 and is mainly expressed in the liver. Its potential binding sites are located in promoter regions and form clusters with binding sites for other transcription factors [33]. The HNF1A protein is composed of 631 amino acids and consists of three functional domains: a dimerization domain (amino acids 1–33), a DNA-binding domain that includes a region necessary for specific DNA recognition (amino acids 100–184) and a homeodomain (amino acids 198–281), and a transactivation domain (amino acids 282–631) [34–37]. HNF1A has been shown to play a critical role in the regulation of AFP gene expression [12, 13]. rs1169288 (Ile27Leu), which is located in the dimerization domain of HNF1A, is essential for DNA-binding by the atypical homeodomain [34]. Speculatively, variation within rs1169288 may influence the ability of HNF1A protein to bind to the AFP gene and change the expression of AFP. Prior studies have shown that HNF1A rs1169288 polymorphism is significantly associated with CRP concentration [18], cardiovascular diseases [21], and decreased *in vitro* transcriptional activity of downstream target gene promoters [38]. However, our study found no significant difference in serum AFP levels among individuals with the different rs1169288 genotypes. A possible explanation is that changes in gene regulation resulting from amino acid variations at this site could not lead to the same degree of change in downstream genes.

Our results showed that healthy Chinese subjects carrying the rs2464196-A allele were more likely to have a low serum level of AFP, and this germline variant also was associated with lower serum AFP levels in HCC patients. Compared with the average serum AFP levels of rs2464196-GG allele carriers, AFP levels in those carrying the AA allele and GA+AA allele were significantly lower. Moreover, according to AFP concentrations used for decision-making regarding HCC diagnosis, the frequency of GA+AA carriers was higher among those with AFP ≤ 20 ng/ml or ≤400 ng/ml than among individuals with AFP > 20 ng/ml or >400 ng/ml. These results showed that the rs2464196-A allele carriers were less likely to have an elevated AFP level, suggesting that this genetic variation site may be useful as a genetic marker for the diagnosis of HCC in the absence of an elevated serum AFP level. rs2464196 (Ser487Asn) is located in the transactivation domain of HNF1A, suggesting that the amino acid change caused by mutation may be associated with decreased transcriptional activity of the AFP gene. Alternatively, the association of HNF1A with AFP level may be due to linkage disequilibrium with other polymorphisms/mutations that are related to AFP gene expression and thus responsible for the associations; the exact mechanisms require further research.

Our data showed a significant linkage disequilibrium between rs2464196 and rs1169310, similar to the results of another study in the Chinese Han population [18] and similar to the results of research in the Arab population [39]. In our study, we observed a significant correlation between rs1169310-T and a decreased serum AFP level, both in healthy individuals and in HCC patients. The rs1169310 is located in the 3′-untranslated region (3′UTR) of the HNF1A gene, and research has shown that the 3′UTR plays a critical role in the posttranscriptional regulation of gene expression by affecting mRNA subcellular localization, stability, and efficiency of translation [40]. Therefore, polymorphisms in this region may influence gene expression and function. Our results showed that the rs1169310-T allele was strongly associated with a lower serum AFP level, indicating that the rs1169310 polymorphism might be a genetic determinant of serum AFP. The exact regulatory mechanism by which the rs1169310 polymorphism affects AFP gene expression needs to be further explored.

The present study has some limitations. First, our results were obtained in a Chinese Han population and, thus, may not apply in other populations. Further research is required to identify possible ethnic differences in the frequency of HNF1A genetic variation. Second, the modest sample size of HCC patients, especially for female patients, may have reduced the statistical power of our analysis. Therefore, large-scale comprehensive studies are needed to further confirm the associations of HNF1A gene polymorphisms with serum AFP levels.

## 5. Conclusion

In summary, our results demonstrated that HNF1Ars2464196 and rs1169310 were associated with serum AFP levels in a healthy Chinese population and Chinese HCC patients. Individuals carrying the HNF1A rs2464196-A allele or rs1169310-T allele tended to have a low serum AFP level. However, no significant associations were observed between HNF1A rs1169288 alleles and the serum AFP concentration. Our findings provide new insights into genetic determinants of the serum AFP levels and promote a more complete understanding of why some HCC patients do not have an elevated AFP level. Detection of HNF1A mutations in patients with a low serum AFP concentration may support more accurate diagnosis of HCC compared with only using AFP concentration.

## Figures and Tables

**Table 1:** Basic characteristics and HNF1A SNP distribution frequencies by gender among healthy Chinese subjects.

	Total population	Men	Women	*P*
Sex (*n*)	1010	615	395	—
Age (years)	48.22 ± 7.80	48.09 ± 7.84	48.44 ± 7.75	0.488
BMI (kg/m^2^)	24.56 ± 2.58	25.33 ± 2.24	23.35 ± 2.61	<0.001
Systolic pressure (mmHg)	116.80 ± 15.34	118.05 ± 14.89	114.86 ± 15.84	0.001
Diastolic pressure (mmHg)	75.74 ± 9.85	77.00 ± 9.96	73.77 ± 9.37	<0.001
LDL (mmol/l)	2.65 ± 0.69	2.65 ± 0.67	2.65 ± 0.71	0.864
HDL (mmol/l)	0.96 ± 0.23	0.88 ± 0.19	1.08 ± 0.24	<0.001
TC (mmol/l)	4.98 ± 0.88	4.93 ± 0.84	5.06 ± 0.92	0.014
AST (U/l)	20.40 (17.40, 24.80)	21.60 (18.70, 26.00)	18.80 (16.50, 22.40)	<0.001
FPG (mmol/l)	5.23 ± 0.90	5.28 ± 1.01	5.16 ± 0.69	0.019
ALT (U/l)	18.50 (13.10, 26.83)	21.50 (15.50, 29.80)	14.40 (10.10, 20.30)	<0.001
TG (mmol/l)	1.27 (0.88, 1.89)	1.46 (1.02, 2.07)	1.04 (0.75, 1.53)	<0.001
GGT (U/l)	21.50 (14.78, 35.73)	27.50 (19.00, 41.70)	15.10 (11.90, 20.90)	<0.001
CRP (mg/l)	1.08 (0.68, 1.81)	1.18 (0.79, 2.03)	0.85 (0.53, 1.52)	<0.001
AFP (ng/ml)	3.61 (2.69, 4.70)	3.48 (2.61, 4.67)	3.07 (2.84, 4.70)	0.158
Fatty liver (%)	44.7	56.1	26.8	<0.001
rs1169288				
AA	311 (30.8%)	189 (30.7%)	122 (30.9%)	0.997
AC	483 (47.8%)	294 (47.8%)	189 (47.8%)	
CC	216 (24.4%)	132 (21.5%)	84 (21.3%)	
rs2464196				
GG	290 (28.7%)	173 (28.1%)	117 (29.6%)	0.355
GA	473 (46.8%)	282 (45.9%)	191 (48.4%)	
AA	247 (24.5%)	160 (26.0%)	87 (22.0%)	
rs1169310				
CC	282 (27.9%)	166 (27.0%)	116 (29.4%)	0.327
CT	478 (47.3%)	287 (46.7%)	191 (48.4%)	
TT	250 (24.8%)	162 (26.3%)	88 (22.3%)	

Data are presented as the mean ± standard deviation (SD) or *n* (%). Nonnormally distributed data are presented as the median (interquartile range (IQR)). The TG, CRP, ALT, AST, GGT, and AFP data were ln-transformed before analysis. *P* value indicates the comparison of male *vs.* female using independent-sample *t*-test for continuous variables and using Chi-squared test for categorical variables and SNP distribution frequencies.

**Table 2:** Association of rs2464196 and rs1169310 genotypes with AFP levels in healthy Chinese subjects.

	rs2464196					rs1169310				
	*R* ^2^	*β*	95% CI	SE	*P*	*R* ^2^	*β*	95% CI	SE	*P*
Model 1	0.017	-0.078	-0.114, -0.043	0.018	0.000018^∗^	0.013	-0.070	-0.106, -0.034	0.018	0.000139^∗^
Model 2	0.028	-0.080	-0.115, -0.044	0.018	0.000012^∗^	0.024	-0.072	-0.108, -0.036	0.018	0.000087^∗^
Model 3	0.066	-0.072	-0.108, -0.037	0.018	0.000056^∗^	0.063	-0.065	-0.100, -0.029	0.018	0.000339^∗^

Model 1: no adjustment; model 2: adjusted for age, gender, and BMI; model 3: adjusted for age, gender, BMI, blood pressure, LDL, HDL, TC, lnAST, lnALT, lnTG, lnGGT, lnCRP, FPG, and fatty liver. *β* represents the unstandardized beta coefficients (*β*) for the effects of HNF1A polymorphisms on the AFP levels. *P* values for the associations of the rs2464196 or rs1169310 genotypes with the AFP level. ^∗^*P* values that passed Bonferroni correction (*P* < 0.0167).

**Table 3:** Clinical features and SNP genotypes among Chinese HCC patients (*n* = 185).

Characteristics	Total (%)
Gender	
Male, *n* (%)	159 (85.9)
Female, *n* (%)	26 (14.1)
Median age (years)	58.2 ± 11.4
<60 years, *n* (%)	106 (57.3)
≥60 years, *n* (%)	79 (42.7)
AFP (ng/ml)	79.82 (7.59, 1996.50)
≤20 ng/ml, *n* (%)	68 (36.8%)
≤400 ng/ml, *n* (%)	118 (63.8%)
ALT (U/l), median (IQR)	37.00 (19.00, 65.85)
AST (U/l), median (IQR)	51.00 (35.00, 89.00)
HBV infection	
Negative, *n* (%)	32 (17.3)
Positive, *n* (%)	153 (82.7)
HCV infection	
Negative, *n* (%)	161 (87.0%)
Positive, *n* (%)	24 (13.0%)
Tumor size	
≤5 cm, *n* (%)	121 (65.4)
>5 cm, *n* (%)	64 (34.6)
Clinical stage	
I, *n* (%)	32 (17.3)
II, *n* (%)	91 (49.2)
III, *n* (%)	29 (15.7)
IV, *n* (%)	33 (17.8)
Tumor differentiation	
I, *n* (%)	35 (18.9)
II, *n* (%)	107 (57.8)
III, *n* (%)	43 (23.2)
rs1169288	
AA, *n* (%)	58 (31.4)
AC, *n* (%)	95 (51.3)
CC, *n* (%)	32 (17.3)
rs2464196	
GG, *n* (%)	42 (22.7)
GA, *n* (%)	98 (53.0)
AA, *n* (%)	45 (24.3)
rs1169310	
CC, *n* (%)	40 (21.6)
CT, *n* (%)	101 (54.6)
TT, *n* (%)	44 (23.8)

**Table 4:** Association of rs2464196 and rs1169310 genotypes with AFP level in Chinese HCC patients.

	rs2464196					rs1169310				
	*R* ^2^	*β*	95% CI	SE	*P*	*R* ^2^	*β*	95% CI	SE	*P*
Model 1	0.037	-1.040	-1.767, -0.314	0.368	0.005260^∗^	0.047	-1.179	-1.915, -0.444	0.373	0.001827^∗^
Model 2	0.256	-0.964	-1.633, -0.294	0.339	0.005012^∗^	0.265	-1.099	-1.778, -0.420	0.344	0.001665^∗^
Model 3	0.586	-0.530	-1.041, -0.018	0.259	0.042404	0.587	-0.576	-1.100, -0.053	0.265	0.031127

Model 1: no adjustment; model 2: adjusted for age and gender, lnALT, lnAST, and HBV and HCV infection status; model 3: adjusted for age, gender, lnALT, lnAST, HBV and HCV infection status, tumor size, clinical stage, and differentiation degree. *β* represents the unstandardized beta coefficients (*β*) for the effects of HNF1A polymorphisms on the AFP levels. *P* value for the association of rs2464196 or rs1169310 genotypes with AFP level. ^∗^*P* values that passed Bonferroni correction (*P* < 0.0167).

**Table 5:** Comparison of genotype frequencies among Chinese HCC patients according to AFP concentrations with the cut − off value > 20 ng/ml.

Genotype	AFP ≤ 20 ng/ml (*n* = 68) *n* (%)	AFP > 20 ng/ml (*n* = 117) *n* (%)	*P* ^1^	*P* ^2^
rs2464196				
GG	8 (11.8)	34 (29.1)	0.007	0.024
GA	42 (61.7)	56 (47.8)	0.068	
AA	18 (26.5)	27 (23.1)	0.604	
GA+AA	60 (88.2)	83 (70.9)	0.007	
rs1169310				
CC	7 (10.3)	33 (28.2)	0.004	0.017
CT	43 (63.2)	58 (49.6)	0.072	
TT	18 (26.5)	26 (22.2)	0.513	
CT+TT	61 (89.7)	84 (71.8)	0.004	

*P* ^1^ represents the difference in genotype frequencies of rs2464196 and rs1169310 between the groups with and without AFP > 20 ng/ml; Chi-squared tests were adopted. *P*^2^ represents the difference in the distribution of rs2464196 and rs1169310 between the two groups; *R* × *C* Chi-squared tests were used.

**Table 6:** Comparison of genotype frequencies between HCC patients according to AFP concentrations with the cut − off value > 400 ng/ml.

Genotype	AFP ≤ 400 ng/ml (*n* = 118) *n* (%)	AFP > 400 ng/ml (*n* = 67) *n* (%)	*P* ^1^	*P* ^2^
rs2464196				
GG	20 (16.9)	22 (32.8)	0.013	0.036
GA	69 (58.5)	29 (43.3)	0.047	
AA	29 (24.6)	16 (23.9)	0.916	
GA+AA	98 (83.1)	45 (67.2)	0.013	
rs1169310				
CC	18 (15.3)	22 (32.8)	0.005	0.018
CT	71 (60.1)	30 (44.8)	0.043	
TT	29 (24.6)	15 (22.4)	0.737	
CT+TT	100 (84.7)	45 (67.2)	0.005	

*P* ^1^ represents the difference in genotype frequencies of rs2464196 and rs1169310 between the groups with and without AFP > 400 ng/ml; Chi-squared tests were adopted. *P*^2^ represents the difference in the distributions of rs2464196 and rs1169310 between the two groups; *R* × *C* Chi-squared tests were used.

## Data Availability

Basic and pathological data and SNP typing data for all healthy subjects and HCC patients used to support the findings of this study are included within the supplementary information files.

## Supplementary Materials

Supplementary Materials Supplementary Table 1: primers and probes used for genotyping HNF1A SNPs. Supplementary Table 2: basic characteristics of apparently healthy subjects compared among the HNF1A genotypes. Supplementary Table 3: HNF1A rs2464196-rs1169310 haplotype frequencies in healthy Chinese subjects and HCC patients. Supplementary Table 4: associations of HNF1A rs2464196-rs1169310 haplotypes with AFP level in healthy Chinese subjects. Supplementary Table 5: associations of HNF1A rs2464196-rs1169310 haplotypes with AFP level in healthy Chinese HCC patients. Supplementary Figure 1: HNF1A SNP genotyping and DNA sequencing results.
