# Supplementary material for: Association of Common Variants in HNF1A Gene with Serum AFP Level in Healthy Chinese Individuals and HCC Patients
Source: Dis Markers. 2019 Nov 7;2019:6273497. doi: 10.1155/2019/6273497 (PMC6935455; doi:10.1155/2019/6273497)
Supplement: Supplementary Materials — Supplementary Table 1: primers and probes used for genotyping HNF1A SNPs. Supplementary Table 2: basic characteristics of apparently healthy subjects compared among the HNF1A genotypes. Supplementary Table 3: HNF1A rs2464196-rs1169310 haplotype frequencies in healthy Chinese subjects and HCC patients. Supplementary Table 4: associations of HNF1A rs2464196-rs1169310 haplotypes with AFP level in healthy Chinese subjects. Supplementary Table 5: associations of HNF1A rs2464196-rs1169310 haplotypes with AFP level in healthy Chinese HCC patients. Supplementary Figure 1: HNF1A SNP genotyping and DNA sequencing results. [file 6273497.f1.pdf]

Supplementary materials

Supplementary Table 1Primers and probes used for genotyping HNF1A SNPs

SNP	Region	Primer name	Sequence (5' to 3')
rs1169288 (A/C)	exon 1	Forward primer A	5'-GGC TGA GCA AAG AGG CAC T*A*A-3'
		Forward primer C	5'-GGC TGA GCA AAG AGG CAC T*A*C-3'
		Reverse primer	5'- CAG GAC TCC CCC TTG TCC AG-3'
		Probe	5'-FAM-CCA GGC ACT GGG TGA GCC GGG GCC- BHQ-1-3'
rs2464196 (G/A)	exon 7	Forward primer G	5'-GCA GAG CCA TGT GAC CCA G*T*G-3'
		Forward primer A	5'-GCA GAG CCA TGT GAC CCA G*T*A-3'
		Reverse primer	5'-GCT CAC CGT GGG GGC TCT-3'
		Probe	5'-FAM-CCC TTC ATG GCC ACC ATG GCT CAG CTG- BHQ-1-3'
rs1169310 (C/T)	3'UTR	Forward primer	5'-GGA CCG CTA CAC CAC TCT G-3'
		Reverse primer C	5'-GAA CAA GGC CAC GCT GAT *A*C-3'
		Reverse primer T	5'-AGA ACA AGG CCA CGC TGA T*A*T-3'
		Probe	5'-FAM-AGC TCA GCA GGT CAC AGC TAC ACA GGC C-BHQ-1-3'

The underlined italic bases near the 3′end of the primers are mismatched to increase the specificity of allele discrimination.

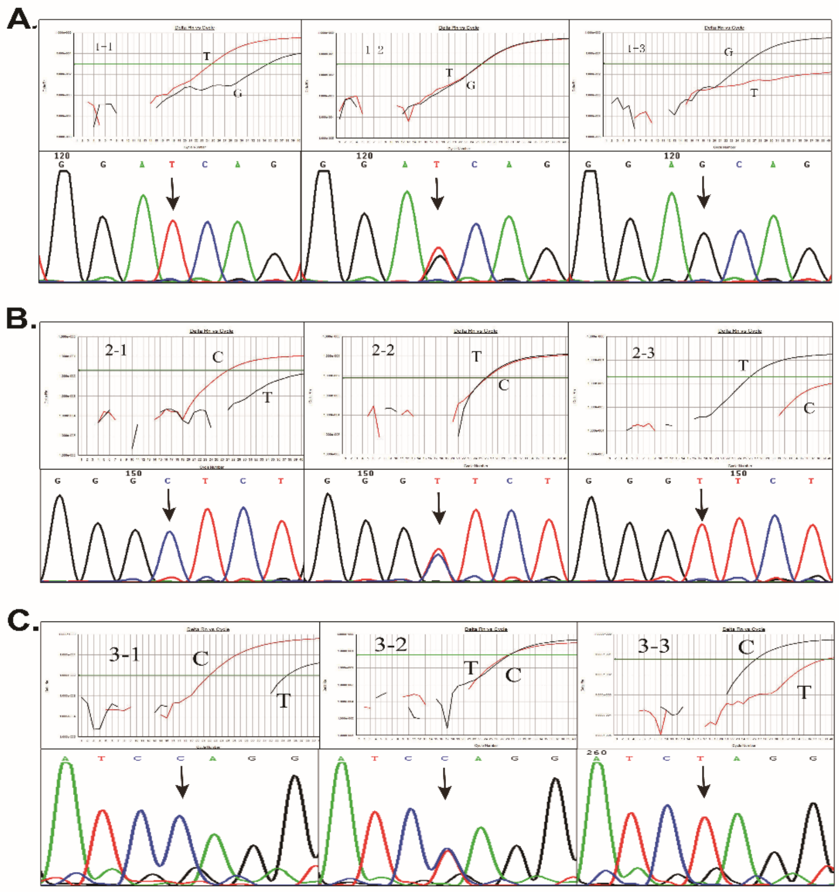

Supplementary Figure 1 HNF1A SNP genotyping and DNA sequencing results. A. Real-time ARMS-PCR amplification plots and sequencing results for HNF1A-rs1169288 (T/G). ARMS-PCR was performed using two reactions. T: Amplification curves of T allele; G: amplification curves of G allele. The different Δ cycle thresholds (Δct= Ct value of T allele minus Ct value of G allele) were used to determine the TT, TG or GG alleles, and the corresponding sequencing results are shown below with black arrows. 1-1: TT genotype, 1-2: TG genotype, 1-3: GG genotype. **B.** Real-time ARMS-PCR amplification plots and sequencing results for HNF1A-rs2464196 (C/T). ARMS-PCR was performed using two reactions. C: Amplification curves of C allele; T: amplification curves of T allele. The different Δ cycle thresholds (Δct= Ct value of C allele minus Ct value of T allele) were used to determine the CC, CT or TT alleles, and

the corresponding sequencing results are shown below with black arrows. 2-1: CC genotype, 2-2: CT genotype, 2-3: TT genotype. **C.** Real-time ARMS-PCR amplification plots and sequencing results for HNF1A-rs1169310 (C/T). ARMS-PCR was performed using two reactions. C: Amplification curves of C allele; T: amplification curves of T allele. The different Δ cycle thresholds (Δct= Ct value of C allele minus Ct value of T allele) were used to determine the CC, CT or TT alleles, and the corresponding sequencing results are shown below with black arrows. 3-1: CC genotype, 3-2: CT genotype, 3-3: TT genotype.

Supplementary Table 2 Basic characteristics of apparently healthy subjects compared among the HNF1A genotypes

	Total	rs1169288				rs2464196				rs1169310			
		AA (n=311)	AC (n=483)	CC (n=216)	*P*	GG (n=290)	GA (n=473)	AA (n=247)	*P*	CC (n=282)	CT (n=478)	TT (n=250)	*P*
Gender (male/female)	1010	189/122	294/189	132/84	0.997	173/117	282/191	160/87	0.355	166/116	287/191	162/88	0.327
Age (years)	48.22±7.80	47.77±0.44	48.31±0.36	48.69±0.51	0.387	47.68±7.63	48.45±7.78	48.43±8.05	0.378	47.55±7.63	48.49±7.79	48.48±8.00	0.230
BMI (kg/m^2)	24.56±2.58	24.63±0.14	24.51±0.12	24.56±0.19	0.828	24.69±2.46	24.42±2.57	24.67±2.74	0.288	24.67±2.50	24.45±2.54	24.66±2.73	0.414
Systolic pressure (mmHg)	116.80±15.34	115.99±0.89	116.79±0.70	118.00±1.03	0.334	117.39±16.27	116.25±14.75	117.17±15.37	0.557	117.18±16.36	116.28±14.75	117.37±15.30	0.589
Diastolic pressure (mmHg)	75.74±9.85	75.11±0.55	75.80±0.46	76.50±0.63	0.280	76.04±10.30	75.62±9.27	75.60±10.42	0.827	75.99±10.12	75.64±9.40	75.64±10.42	0.879
LDL (mmol/L)	2.65±0.69	2.69±0.04	2.64±0.03	2.62±0.05	0.437	2.70±0.68	2.66±0.68	2.57±0.70	0.076	2.72±0.70	2.65±0.67	2.57±0.70	0.054
HDL (mmol/L)	0.96±0.23	0.96±0.01	0.95±0.01	0.96±0.02	0.824	0.97±0.26	0.96±0.22	0.94±0.23	0.282	0.97±0.26	0.96±0.22	0.94±0.23	0.255
TC (mmol/L)	4.98±0.88	5.00±0.05	4.97±0.04	4.97±0.06	0.890	5.04±0.89	4.97±0.86	4.92±0.88	0.275	5.06±0.91	4.97±0.85	4.92±0.88	0.172
AST (U/L)	20.40 (17.40, 24.80)	20.50 (17.50, 25.00)	20.50 (17.60, 25.20)	20.30 (16.90, 24.68)	0.189	20.85 (17.88, 25.70)	20.10 (17.30, 24.30)	20.60 (17.60, 25.70)	0.056	20.85 (17.88, 25.35)	20.15 (17.27, 24.33)	20.60 (17.60, 25.63)	0.107
FPG (mmol/L)	5.23±0.90	5.17±0.05	5.26±0.04	5.26±0.06	0.376	5.21±0.99	5.23±0.81	5.27±0.93	0.792	5.23±1.00	5.22±0.81	5.26±0.93	0.832
ALT (U/L)	18.50 (13.10, 26.83)	18.50 (12.80, 26.9)	18.60 (13.40, 27.9)	17.40 (12.40, 25.1)	0.199	19.25 (13.40, 27.40)	18.10 (13.00, 25.60)	18.80 (13.10, 27.20)	0.165	19.15 (13.33, 27.40)	18.15 (13.00, 26.10)	18.95 (13.10, 26.98)	0.333
TG (mmol/L)	1.27 (0.88, 1.89)	1.28 (0.89, 1.90)	1.24 (0.85, 1.89)	1.31 (0.88, 1.86)	0.843	1.28 (0.90, 1.97)	1.23 (0.84, 1.82)	1.33 (0.91, 1.93)	0.067	1.28 (0.86, 1.94)	1.23 (0.84, 1.82)	1.33 (0.91, 1.94)	0.092
GGT (U/L)	21.50 (14.78, 35.73)	22.70 (14.90, 36.20)	21.30 (14.70, 36.80)	21.00 (14.68, 33.40)	0.368	23.70 (15.18, 36.95)	20.70 (14.10, 35.75)	21.40 (15.50, 33.60)	0.190	23.10 (15.05, 36.33)	20.85 (14.10, 36.35)	21.40 (15.48, 34.15)	0.320
CRP (mg/L)	1.08 (0.68, 1.81)	1.13 (0.72, 1.95)	1.08 (0.69, 1.78)	0.98 (0.57, 1.58)	0.023	1.11 (0.67, 2.09)	1.05 (0.67, 1.74)	1.06 (0.68, 1.61)	0.276	1.12 (0.70, 2.09)	1.05 (0.67, 1.73)	1.06 (0.69, 1.67)	0.143
Fatty liver (%)	44.70	43.40	45.30	44.90	0.864	43.10	44.20	47.40	0.588	43.30	44.10	47.20	0.629

Data are presented as mean ± standard deviation (SD) or n (%). Non-normally distributed data are presented as median (interquartile range [IQR]). The TG, CRP, ALT, AST and GGT data were ln-transformed before analysis. *P* value indicates the comparison among the genotypes of each SNP locus of HNF1A using ANOVA for continuous variables and Chi-square test for categorical variables and SNP distribution frequencies.

Supplementary Table 3 HNF1A rs2464196-rs1169310 haplotype frequencies in healthy Chinese subjects and HCC patients.

	rs2464196	rs1169310	Frequency (%)	
			Healthy subjects	HCC patients
H1	G	C	50.98	48.65
H2	G	T	1.15	0.54
H3	A	C	0.60	0.27
H4	A	T	47.27	50.54

Supplementary Table 4 Associations of HNF1A rs2464196 and rs1169310 haplotypes with AFP level in healthy Chinese subjects.

	R^2	Haplotype	β	95% CI	SE	*P*
Model 1	0.010	GC	0			
		AT	-0.080	-0.117, -0.043	0.019	<0.001
		Other	0.072	-0.071, 0.214	0.072	0.324
Model 2	0.023	GC	0			
		AT	-0.081	-0.118, -0.044	0.019	<0.001
		Other	0.069	-0.072, 0.21	0.072	0.339
Model 3	0.073	GC	0			
		AT	-0.074	-0.111, -0.038	0.018	<0.001
		Other	0.056	-0.083, 0.194	0.071	0.430

Model 1: no adjustment; Model 2: adjusted for age, gender, and BMI; Model 3: adjusted for age, gender, BMI, blood pressure, HDL, TC, lnAST, lnALT, lnTG, lnGGT, lnCRP, FPG, and fatty liver. β means the unstandardized beta coefficients (β) for the effects of HNF1A polymorphisms on the AFP levels. *P* values for the associations of the haplotypes of rs2464196 and rs1169310 with the lnAFP level in healthy subjects.

Supplementary Table 5 Associations of HNF1A rs2464196- rs1169310 haplotypes with AFP level in Chinese HCC patients.

	R2	Haplotype	β	95% CI	SE	*P*
Model 1	0.022	GC	0			
		AT	-1.033	-1.744, -0.321	0.362	0.005
		Other	-0.437	-4.403, 3.529	2.017	0.829
Model 2	0.073	GC	0			
		AT	-0.899	-1.6, -0.199	0.356	0.012
		Other	-0.432	-4.306, 3.441	1.970	0.826
Model 3	0.603	GC	0			
		AT	-0.474	-0.948, 0	0.241	0.049
		Other	-0.477	-3.046, 2.092	1.306	0.715

Model 1: no adjustment; Model 2: adjusted for age and gender; Model 3: adjusted for age, gender, lnALT, lnAST, HBV and HCV infection status, tumor size, clinical stage, and differentiation degree. β means the unstandardized beta coefficients (β) for the effects of HNF1A polymorphisms on the AFP levels. *P* values for the associations of the haplotypes of rs2464196 and rs1169310 with the lnAFP level in HCC patients.
